# Prevalence of glucose-6-phosphate dehydrogenase (G6PD) deficiency among malaria patients in Upper Myanmar

**DOI:** 10.1186/s12879-018-3031-y

**Published:** 2018-03-16

**Authors:** Jinyoung Lee, Tae Im Kim, Jung-Mi Kang, Hojong Jun, Hương Giang Lê, Thị Lam Thái, Woon-Mok Sohn, Moe Kyaw Myint, Khin Lin, Tong-Soo Kim, Byoung-Kuk Na

**Affiliations:** 1 Department of Parasitology and Tropical Medicine, and Institute of Health Sciences, Gyeongsang National University College of Medicine, Jinju, 52727 Republic of Korea; 2 BK21Plus Team for Anti-aging Biotechnology and Industry, Department of Convergence Medical Science, Gyeongsang National University, Jinju, 52727 Republic of Korea; 3 Department of Tropical Medicine, and Inha Research Institute for Medical Sciences, Inha University College of Medicine, Incheon, 22212 Republic of Korea; 4 Department of Medical Research Pyin Oo Lwin Branch, Pyin Oo Lwin, Myanmar; 5 Present address: Department of Tropical Medicine, and Inha Research Institute for Medical Sciences, Inha University College of Medicine, Incheon, 22212 Republic of Korea; 6 Present address: Planning and Management Division, Nakdonggang National Institute of Biological Resources, Sangju, 37242 Republic of Korea

**Keywords:** Glucose-6-phosphate dehydrogenase (G6PD), G6PD deficiency, Malaria, Primaquine, Myanmar

## Abstract

**Background:**

Glucose-6-phosphate dehydrogenase (G6PD; EC 1.1.1.49) deficiency is one of the most common X-linked recessive hereditary disorders in the world. Primaquine (PQ) has been used for radical cure of *P. vivax* to prevent relapse. Recently, it is also used to reduce *P. falciparum* gametocyte carriage to block transmission. However, PQ metabolites oxidize hemoglobin and generate excessive reactive oxygen species which can trigger acute hemolytic anemia in malaria patients with inherited G6PD deficiency.

**Methods:**

A total of 252 blood samples collected from malaria patients in Myanmar were used in this study. G6PD variant was analysed by a multiplex allele specific PCR kit, DiaPlexC™ G6PD Genotyping Kit [Asian type]. The accuracy of the multiplex allele specific PCR was confirmed by sequencing analysis.

**Results:**

Prevalence and distribution of G6PD variants in 252 malaria patients in Myanmar were analysed. Six different types of G6PD allelic variants were identified in 50 (7 females and 43 males) malaria patients. The predominant variant was Mahidol (68%, 34/50), of which 91.2% (31/34) and 8.8% (3/34) were males and females, respectively. Other G6PD variants including Kaiping (18%, 9/50), Viangchan (6%, 3/50), Mediterranean (4%, 2/50), Union (2%, 1/50) and Canton (2%, 1/50) were also observed.

**Conclusions:**

Results of this study suggest that more concern for proper and safe use of PQ as a radical cure of malaria in Myanmar is needed by combining G6PD deficiency test before PQ prescription. Establishment of a follow-up system to monitor potential PQ toxicity in malaria patients who are given PQ is also required.

## Background

Glucose-6-phosphate dehydrogenase (G6PD; EC 1.1.1.49) is an enzyme that maintains redox equilibrium in cells by catalyzing the first reaction in the pentose phosphate pathway. This pathway is the single source to supply reduced nicotinamide adenine dinucleotide phosphate (NADPH) which confers primary oxidative defense in red blood cells (RBCs). Therefore, G6PD activity is crucial to protect RBCs from oxidative stresses induced by free radicals derived from oxygen and organic compounds such as drugs and their metabolites [1]. G6PD is encoded by *G6PD* gene that spans 18 kb on the X chromosome (Xq28), consisting of 13 exons separated by 12 introns [2]. Point mutations of this gene result in different levels of G6PD activity, consequently causing a diverse range of biochemical and clinical phenotypes [1]. More than 400 G6PD variants have been identified up to date, of which 186 variants are linked to G6PD deficiency by decreasing the activity or stability of G6PD [1, 3, 4]. Clinical manifestations caused by these G6PD variants vary from severe to asymptomatic and G6PD deficiency can be usually assessed by enzymatic and/or genetic assays.

G6PD deficiency is one of the most common X-linked recessive hereditary disorders in the world. It is characterized by abnormally low levels of G6PD [5, 6]. G6PD deficient RBCs are more vulnerable to damages caused by oxidative stresses induced by foods and drugs, leading to acute hemolytic anemia (AHA) [7]. Most individuals with G6PD deficiency are clinically asymptomatic, but some G6PD variants could be lethal due to complete loss of enzyme activity. G6PD deficiency has been recognized as a good example of natural selection since strong geographic correlation of G6PD deficiency distribution with historical endemicity patterns of malaria has been identified [8–11]. G6PD deficiency provides resistance against severe malaria [12–15]. The precise mechanism of G6PD deficiency for protective effect against severe malaria is not fully understood, but it is likely to be associated with the effects of G6PD deficiency on RBC physiology. Optimum redox status in RBCs is essentially required by malaria parasites for their survival and development in RBCs. Inability to maintain normal redox status in G6PD deficient RBCs results in oxidative stress that may unfavorable to malaria parasites, which supporting the protection hypothesis [13]. But paradoxically, G6PD deficiency is also a stumbling block in fighting against malaria. Primaquine (PQ) has been used for radical cure of *Plasmodium vivax* to prevent relapse. It is recently being used to reduce *P. falciparum* gametocyte carriage to block transmission. However, PQ metabolites can oxidize hemoglobin and generate excessive reactive oxygen species which can cause lethal AHA in malaria patients with inherited G6PD deficiency [16, 17]. Due to this reason, the WHO recommends G6PD test prior to administration of PQ for radical cure of *P. vivax* [18]. PQ has been widely used in Myanmar for radical cure of *P. vivax* and *P. ovale*. Using single dose PQ as a gametocytocide for *P. falciparum* is also recommended. However, diagnosis of G6PD status in malaria patients before PQ prescription is not generally conducted in the country. Information on G6PD deficiency and potential risk of PQ by the genetic disorder in malaria patients are also lacking.

Several studies have reported the prevalence of G6PD deficiency and its variants in Myanmar population [19–23]. However, the status of G6PD deficiency in malaria patients has not been reported in the literature. The aim of this study was to determine the prevalence of G6PD deficiency in confirmed malaria patients in Upper Myanmar. A total of 50 out of 252 enrolled malaria patients were determined to have G6PD deficiency with several different genetic variants. Our results strongly suggest the necessity to monitor the presence of G6PD deficiency in Myanmar malaria patients before prescribing PQ.

## Methods

### Blood samples

A total of 252 blood samples were used in this study (Table 1). These blood samples were collected from malaria patients who attended public health centers in Naung Cho, Pyin Oo Lwin, Tha Beik Kyin townships, and Mandalay in Upper Myanmar from August 2013 to December 2015. The average age of these 252 subjects was 27.8 years (range, 14–53 years). Malaria infections were confirmed by microscopic examination and species-specific nested polymerase chain reaction (PCR) [24]. Informed written consent was obtained from each enrolled patient prior to blood sampling. Two or three drops of blood (approximately 50 μl) from a finger prick were blotted onto Whatman 3 MM filter paper (GE Healthcare, Maidstone, UK) and allowed to air dry. This study was approved by the Ethics Review Committee, Department of Medical Research, Myanmar (1/Ethics/DMRUM/2013 and 97/Ethics 2015) and by the Ethical Review Committee of Inha University School of Medicine, Korea (INHA 15–013).

**Table 1:** Blood samples from malaria patients used in this study

	*P. falciparum*	*P. vivax*	*P. falciparum + P. vivax*	*Total*
Male	92	85	38	215
Female	14	14	9	37
Total	106	99	47	252

### G6PD multiplex allele specific PCR

Genomic DNA was extracted from *Plasmodium*-positive blood filters using QIAamp DNA Blood Mini Kit (Qiagen, Hilden, Germany) according to the manufacturer’s protocols. G6PD variants were screened using a multiplex allele specific PCR kit, DiaPlexC™ G6PD Genotyping Kit [Asian type] (SolGent, Daejeon, Korea). This kit can detect eight different G6PD variants: Vanua Lava (383 T > C, 154 bp), Mahidol (487 G > A, 337 bp), Mediterranean (563 C > T, 262 bp), Coimbra (592 C > T, 243 bp), Viangchan (871 G > A, 501 bp), Union (1360 C > T, 803 bp), Canton (1376 G > T, 681 bp), and Kaiping (1388 G > A, 557 bp). Multiplex PCR was performed in a 25 μl reaction containing 3 μl of genomic DNA (approximately 20 ng), 12.5 μl of 2× multiplex PCR Smart mix, 2 μl of primer mixture and 7.5 μl of distilled water. Amplification was performed with a denaturation at 95 °C for 15 min followed by amplification steps of 35 cycles (95 °C for 30 s, 60 °C for 30 s and 72 °C for 40 s) and a final extension at 72 °C for 5 min. Each amplicon was analyzed on 3% agarose gel to check size of amplified PCR product. Each reaction was confirmed with an internal control (wild type G6PD control, 1234 bp) following the protocols provided by the manufacturer.

### Cloning and DNA sequencing analysis of G6PD variants

To confirm the accuracy of the multiplex allele specific PCR and clarify if there were any other mutations besides the ones identified by the PCR method, samples for each variant detected by the multiplex method were randomly selected and subjected to nucleotide sequencing analysis. Variant regions corresponding to exon 6, exon 9, exon 11, and exon 12 of G6PD gene were amplified from each genomic DNA using primers described previously [25]. To minimize nucleotide mismatch in sequences during PCR amplification, Ex Taq DNA polymerase (Takara, Otsu, Japan) with proofreading activity was used for all PCR procedures. Each PCR product was cloned into T&A cloning vector (Real Biotech Corporation, Banqiao City, Taiwan) and transformed into *Escherichia coli* DH5α competent cells. Positive clones with appropriate insert were selected. Nucleotide sequences of cloned G6PD regions were determined by automatic DNA sequencing. At least two or three clones from each sample were sequenced to ensure sequencing accuracy. These obtained nucleotide sequences were then analyzed using EditSeq and SeqMan in DNASTAR package (DNASTAR, Madison, WI, USA).

## Results

**Fig. 1:**
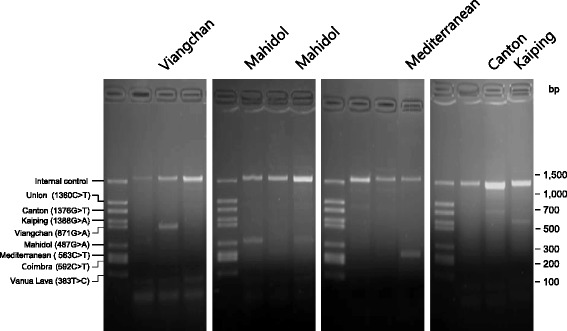
G6PD multiplex allele specific PCR. G6PD variants in 252 Myanmar malaria patients were analyzed by multiplex allele specific PCR. Six different types of G6PD variants were identified in 50 malaria patients (19.8%): Mahidol (487 G > A, 337 bp), Mediterranean (563 C > T, 262 bp), Viangchan (871 G > A, 501 bp), Union (1360 C > T, 803 bp), Canton (1376 G > T, 681 bp) and Kaiping (1388 G > A, 557 bp). Representative PCR results for G6PD variants are presented. Each PCR reaction was confirmed by an internal control (wild type G6PD control, 1234 bp) following the manufacturer’s instruction. The results presented in this figure were obtained in male malaria patients except for Canton

**Fig. 2:**
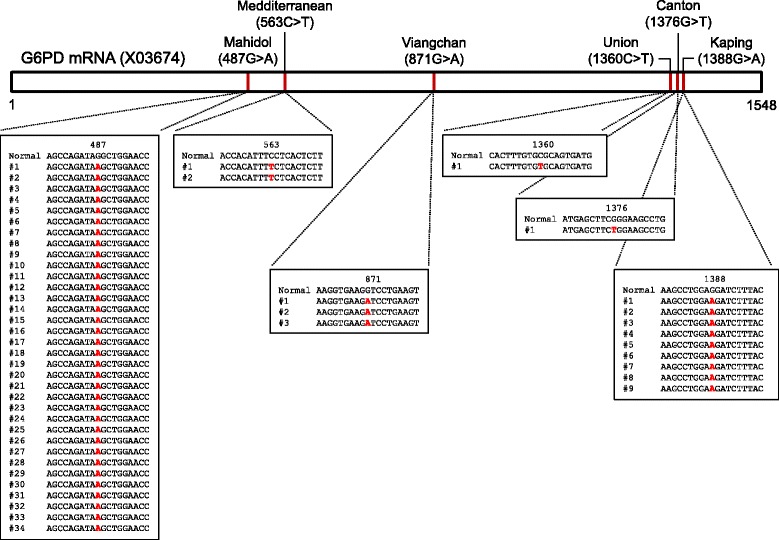
Schematic diagram of G6PD variants in Myanmar malaria patients. The 50 G6PD variants identified by multiplex allele specific PCR were confirmed by sequencing analysis

A total of 252 blood samples from Myanmar malaria patients were analyzed to estimate the frequency of G6PD deficiency and its variants in these patients. The G6PD multiplex allele specific PCR analysis of 252 blood samples revealed several types of G6PD variants in 50 malaria patients (Fig. 1). Although several weak non-specific amplification products were observed in some blood samples, amplification products were well corresponded to one of G6PD variants in the 50 blood samples. To determine whether these were non-specifically amplified products or real G6PD variants, variant regions of G6PD gene were amplified from the 50 samples and subsequently sequenced. Sequencing analyses confirmed that all 50 samples had corresponding G6PD variants consistent with multiplex allele specific PCR results (Fig. 2). Multiplex allele specific PCR and sequencing analysis revealed six different G6PD allelic variants in the 50 blood samples. Mahidol variant (68%, 34/50) was the most common G6PD mutation identified, followed by Kaiping (18%, 9/50), Viangchan (6%, 3/50), Mediterranean (4%, 2/50), Union (2%, 1/50), and Canton (2%, 1/50) variants. Coimbra or Vanua Lava G6PD variant was not detected. The overall frequency of G6PD deficiency was approximately 19.9% (50/252), among which 20.0% (43/215) and 18.9% (7/37) were males and females, respectively (Table 2). Of these 50 malaria patients with G6PD variants, 25 (50.0%), 17 (34.0%), and 8 (16.0%) were infected with *P. falciparum*, *P. vivax*, and co-infections with both *P. falciparum* and *P. vivax*, respectively.

**Table 2:** Frequency of G6PD deficiency and G6PD variants in Myanmar malaria patients

	Amino acid substitution	WHO classification^a^	Female	Male	Total (%)
Normal			30	172	202 (80.1)
G6PD deficiency			7	43	50 (19.9)
Vanua Lava (383T>C)	L128P	II	0	0	0 (0.0)
Mahidol (487G>A)	G163S	III	3	31	34 (13.5)
Mediterranean (563C>T)	S188F	II	1	1	2 (0.8)
Coimbra (592C>T)	R198C	II	0	0	0 (0.0)
Viangchan (871G>A)	V291M	III	0	3	3 (1.2)
Union (1360C>T)	R454C	II	0	1	1 (0.4)
Canton (1376G>T)	R459L	III	1	0	1 (0.4)
Kaiping (1388G>A)	R463H	II	2	7	9 (3.6)

^a^Class I, severe deficiency (<10% activity) with chronic (nonspherocytic) hemolytic anemia; class II, severe deficiency (<10% activity) with intermittent hemolysis severe deficiency; class III, mild deficiency (10-60% activity) hemolysis with stressors only; class IV, non-deficient, variant, no clinical sequelae (50-150% activity); Class V: Increased enzyme activity, no clinical sequelae (150>% activity)

## Discussion

Although estimated malaria cases have declined remarkably in recent years, Myanmar still has the greatest malaria incidence in the Greater Mekong Region [17]. About 60% of the population (more than 30 million people) resides in malaria endemic areas. More than 350,000 confirmed malaria cases were reported in Myanmar in 2013 [17]. Malaria patients usually receive anti-malaria drugs for treatment in public health care facilities or private sectors. However, G6PD deficiency has not been generally checked for patients. PQ is an anti-malaria drug commonly prescribed either for antirelapsing vivax malaria or for blocking falciparum malaria transmission in Myanmar. However, it can trigger life-threatening acute hemolytic complications in G6PD deficiency individuals. Therefore, information on G6PD deficiency status in malaria patients would be necessary for the establishment of a national malaria control program in Myanmar on anti-malaria drug policy and further research on the safety of PQ.

Fifty out of 252 malaria patients (215 males and 37 females) analyzed in this study had at least one of G6PD variants. Mahidol variant (68%) was the most common G6PD variant identified followed by Kaiping (18%), Viangchan (6%), Mediterranean (4%), Union (2%), and Canton (2%) variants. This result agreed with previous studies on G6PD variants in Myanmar showing that G6PD Mahidol was the most common G6PD variant type, even though the prevalence of G6PD variants among Myanmar population varied in different regions of the country according to ethnic variation [19, 20, 22]. The overall percentage of *P. falciparum* infected individuals with G6PD variants (25/50, 50.0%) and *P. falciparum* infected individuals with non-G6PD variants (81/202, 40.1%) did not differ significantly. When including *P. vivax* mixed infection cases, *P. falciparum* infection rate in individuals with G6PD variants was 66.0% (33/50) while *P. falciparum* infection rate in those with non-G6PD variants was 59.4% (120/202). This result is consistent with a previous report showing that G6PD deficiency may not effectively protect patients against *P. falciparum* infection [26]. Interestingly, no significant association was observed between gender and G6PD variant status in patients with malaria. The frequency of G6PD variants in male (43/215, 20.0%) was similar to that in female (7/37, 18.9%). G6PD deficiency is an X-linked hereditary disease. Therefore, males are usually affected more frequently than females as males only have one X-chromosome [26, 27]. It has been proposed that G6PD variants render males substantial protection against severe malaria [15]. The number of female malaria patients enrolled in this study was smaller than that of males. However, when the incidence of G6PD variants in females was predicted by Hardy-Weinberg model, the estimated value accounted for about 36%. Analysis of the effect of G6PD variant or deficiency on disease severity would be highly interesting. However, all patients enrolled in this study were uncomplicated cases with only typical malaria symptoms. Therefore, it was unable to clearly estimate the direct association between G6PD deficiency and the degree of severity.

This study has another limitation in that G6PD enzyme activity in malaria patients could not be checked due to technical limitation in performing enzyme assay in the field using fresh blood samples. Diagnosis of G6PD deficiency based on enzyme activity is usually cumbersome. Routine laboratory assays including spectrophotometry assay [28] and cytochemical staining assay [29] are not suitable for point-of-care test since these methods require well-equipped laboratories and trained technicians. Fluorescent spot test (FST) is the most widely used alternative assay for G6PD deficiency due to its simplicity and low cost [30]. In this study, FST assay was performed for dried blood filters. However, no reliable results were obtained probably due to poor quality of blood samples which might be due to inadequate storage and transport process. It is known that G6PD activity is influenced by the mutation position occurred in the gene. Correlations between clinical phenotypes and altered G6PD activity due to mutations have been well studied [31–35]. Based on the level of enzyme activity, World Health Organization has classified G6PD variants into five classes: *Class I,* severe deficiency of the enzyme with chronic non-spherocytic hemolytic anemia; *Class II,* severe deficiency with enzyme activity < 10% of normal; *Class III,* moderate deficiency with enzyme activity 10–60% of normal; *Class IV,* very mild to none deficiency with enzyme activity 60–100% of normal; and *Class V,* increased enzyme activity [36]. Among variants detected in this study, Union, Mediterranean, and Kaiping are classified as Class II variants while Mahidol, Viangchan, and Canton are classified as Class III variants [37]. Clinical impact of anti-malaria drugs other than PQ in individuals with different G6PD variants is currently unclear. However, G6PD genotype or phenotype is unlikely to influence the anti-malarial efficacy of other anti-malaria drugs, including combination therapy with artemisinin-based drugs [38, 39]. There is no evidence that PQ shows different pharmacokinetics between normal malaria patients and G6PD deficiency malaria patients [40]. A single dose of PQ 45 mg and/or weekly for eight weeks has been routinely used to treat patients with *P. falciparum* gametocytes and/or *P. vivax* in Myanmar, ignoring G6PD deficiency. However, considering individuals with Class II or III G6PD deficiency have potential risk associated with clinically significant AHA during treatment with PQ, results of this study suggest that more caution is needed when prescribing PQ and other 8-aminoquinoline based anti-malaria drugs for malaria patients. It is also worthy to mention that only several major Asian type G6PD variants are screened in this study. However, considering some G6PD variants are linked to other mutations including silence mutations in the *G6PD* gene [41–43], more systematic analysis of G6PD variants in a larger number of blood samples from malaria patients is needed for in-depth understanding of G6PD deficiency status in Myanmar malaria patients. Nonetheless, results of this study provide valuable information on G6PD deficiency status in malaria patients in Myanmar.

## Conclusions

This study is the first report on the frequency of G6PD deficiency associated with malaria in Myanmar. The overall frequency of G6PD deficiency obtained in this study was similar to or slightly higher than the average expected frequencies for the Myanmar population [19, 20, 22]. *P. falciparum* and *P. vivax* accounted for most of malaria cases in areas where this study was performed. However, it is likely that *P. vivax* is becoming predominant species in these areas with recent decrease of falciparum malaria cases [24]. Therefore, proper and safe use of PQ as a radical cure of *P. vivax* combining with G6PD deficiency test is needed. Establishment of follow-up system to monitor possible PQ toxicity in malaria patients who are taking PQ is also required. Recently, availability of G6PD rapid diagnostic tests (RDT) has been suggested for point-of-care detection of G6PD deficiency in the field [23, 44–49]. RDT can be a promising option for G6PD deficiency screening tool prior to prescribe PQ or other 8-aminoquinoline based anti-malaria drugs that can trigger AHA.

## Abbreviations

AHA: Acute hemolytic anemia
FST: Fluorescent spot test
G6PD: Glucose-6-phosphate dehydrogenase
NADPH: Nicotinamide adenine dinucleotide phosphate
PCR: Polymerase chain reaction
PQ: Primaquine
RBCs: Red blood cells
RDT: Rapid diagnostic tests
